# Differences in Knowledge and Perspectives on the Usage of Artificial Intelligence Among Doctors and Medical Students of a Developing Country: A Cross-Sectional Study

**DOI:** 10.7759/cureus.21434

**Published:** 2022-01-19

**Authors:** Rohin Kansal, Ashvind Bawa, Arpit Bansal, Shubam Trehan, Kashish Goyal, Naresh Goyal, Kashish Malhotra

**Affiliations:** 1 Department of General Surgery, Dayanand Medical College and Hospital, Ludhiana, IND; 2 Department of Internal Medicine, Narayana Medical College, Nellore, IND; 3 Department of Internal Medicine, Dayanand Medical College and Hospital, Ludhiana, IND; 4 Department of Research and Development, Delhi Heart Institute and Multispeciality Hospital, Bathinda, IND; 5 Department of Cardiology, Delhi Heart Institute and Multispeciality Hospital, Bathinda, IND

**Keywords:** artificial intelligence, gender disparity, medical training, medical education, ai

## Abstract

Introduction

Rapid advancements are being made in the field of Artificial Intelligence (AI) to support digital healthcare transformation and provide evidence-based care. The aim of this cross-sectional study was to evaluate the knowledge of basic principles, limitations, and applications of AI in healthcare among medical students and doctors of a developing country.

Methods

Two free webinars were hosted for doctors and medical students in northern India (Punjab state) to create awareness about the role of AI in healthcare and the recent advancements made in various medical specialties. The delegates’ perceptions about their knowledge and interest in AI were ascertained using the Likert scale (1 = low, 5 = high) in the post-event questionnaire. Using Chi-square and cross-tabulation analysis, associations were examined between knowledge of AI, gender, medical experience, and other variables.

Results

Out of the total of 621 registrants, 367 filled the post-event questionnaire and were included in the analysis. Although the majority felt that AI will play an important role in delivering healthcare services in the future (74.4%), they did not feel knowledgeable about the applications (79.6%) and limitations of AI (82.8%). A relatively lesser proportion of doctors (51.6%) felt interested to learn more about AI than medical students (69.3%). Furthermore, a lesser proportion of doctors (65.2%) felt that AI will be beneficial for their career as a doctor as compared with medical students (84.4%). The majority of medical students (83.5%) had never attended any webinar/lecture or course on AI in healthcare and felt that they have received minimal advice (80.7%) from their medical school on teaching about AI and its applications. A significantly (P = 0.001) higher proportion of female medical students were unknowledgeable about the principles and applications of AI than male respondents. However, female medical students were significantly (P = 0.004) more interested than male medical students to learn about AI.

Conclusions

Formal training courses to teach about AI should be focused on to facilitate coherent and scientifically supported dissemination of knowledge in medical schools and hospitals. Further large-scale studies are needed to understand the perception and attitude of medical students and doctors regarding AI to steer policy development and medical education curriculum changes to spark an interest in emerging technologies and drive innovation.

## Introduction

Artificial Intelligence (AI) is an umbrella term used to describe the multidisciplinary approach to use statistical, mathematical, and computer sciences to simulate intelligent behavior [1,2]. AI technologies have rapidly progressed in the field of healthcare and have been able to solve numerous challenges faced while delivering medical services. Rapid advancements are being made in the field of AI to support digital healthcare transformation and provide evidence-based care [3].

Using the various types of AI, the healthcare sector has been aided with tools to help in administration, diagnosis making, treatment planning, medical education, medical record mining, drug creation, and triage assessment [4-7]. However, there are various challenges and limitations of AI that need to be considered before formulating and interpreting the results. Protection of patient’s privacy, ethical concerns, black box problem, reliability of input data, confounders, and adversarial attacks are some of the challenges that need to be addressed [8]. Hence, it becomes important that doctors are well aware of the opportunities and challenges linked to AI to make an informed decision and provide holistic care [9,10].

Medical students are the future of healthcare, and it is important to disseminate the correct scientific principles of emerging technologies in healthcare to drive innovation [5]. Currently, limited curricular and extracurricular learning opportunities are available for medical students to learn about AI [11]. Furthermore, AI-based tools may help improve the healthcare systems in lower middle-income countries [12,13]. This is important because an international report found that over 65% of the low- and lower middle-income countries have reduced their spending on public education due to the COVID-19 pandemic [14].

The aim of this cross-sectional study was to evaluate the knowledge of basic principles, limitations, and applications of AI in healthcare among medical students and doctors of a lower middle-income country.

## Materials and methods

Two free webinars were hosted for medical students and doctors in northern India (Punjab state) to create awareness about the role of AI in healthcare and the recent advancements made in the various medical specialties using AI algorithms. The overarching aim of the session was to spark an interest in AI and encourage healthcare professionals to widen their scope in science and technology. The webinars covered the basic functioning of AI, types of AI, clinical scenarios and applications of AI, limitations of AI, and future career prospects as an AI researcher. A dedicated question-and-answer session was also conducted at the end of the webinar where the doubts of participants were discussed.

A post-webinar questionnaire (Table 1) was distributed to know the perceptions of the delegates about AI. Responses were collected until one week after the webinar date. The questionnaire was circulated using Google Forms, which stores data by data encryption technology securely. The ethics committee of Delhi Heart Institute and Multispeciality Hospital issued approval DHIMH/EC/2021/17, and participation was entirely voluntary. No incentive of any kind was given to fill the survey. No personal identifying details were collected, and informed consent was taken. Our study fully complies with the Declaration of Helsinki.

**Table 1:** Post-webinar questionnaire This questionnaire included 12 questions to ascertain social demographics and the knowledge and experience of medical students and doctors about Artificial Intelligence and its applications. The included options for the corresponding questions have been separated with “OR” in this table.

Questions	Responses
Gender	Male OR Female OR Others – Please Mention
Level of study	First-year medical student OR Second-year medical student OR Third-year medical student (pre-final year student) OR Fourth-year medical student (final year student) OR Doctor
Have you ever previously attended any webinar/lecture/course on Artificial Intelligence in healthcare?	Yes OR No
To what extent do you agree or disagree with the statement “Artificial Intelligence will play an integral role in delivering healthcare services in the future”?	Likert Scale 1–5: Strongly Disagree to Strongly Agree
How knowledgeable do you feel about the basic principles of Artificial Intelligence technology and its applications in healthcare?	Likert Scale 1–5: Extremely Unknowledgeable to Extremely Knowledgeable
How knowledgeable do you feel about the limitations of Artificial Intelligence?	Likert Scale 1–5: Extremely Unknowledgeable to Extremely Knowledgeable
To what extent do you agree or disagree with the statement “Knowledge of Artificial Intelligence will be beneficial for your career as a doctor?”	Likert Scale 1–5: Strongly Disagree to Strongly Agree
Which resource have you used the most to learn about Artificial Intelligence and its applications?	Websites OR Friends and colleagues OR Journal articles and books OR Formal training (e.g., courses) in Artificial Intelligence OR Medical school lectures OR Webinars OR Nothing OR Others – Please Mention
How interested do you feel about learning the principles of Artificial Intelligence and its applications in healthcare?	Likert Scale 1–5: Extremely Uninterested to Extremely Interested
To what extent do you agree or disagree with the statement “Formal training is needed in medical schools and hospitals to teach about Artificial Intelligence and its applications in healthcare”?	Likert Scale 1–5: Strongly Disagree to Strongly Agree
To what extent has your medical school or hospital taught you about Artificial Intelligence and its applications?	Likert Scale 1–5: Minimal Advice to Extensive Advice
To what extent do you agree or disagree with the statement “Artificial Intelligence may replace physicians in some specialties in the future”?	Likert Scale 1–5: Strongly Disagree to Strongly Agree

The delegates’ opinions about their knowledge and interests in AI were ascertained using the Likert scale (1 = low, 5 = high) in this questionnaire. Using Chi-square and cross-tabulation analysis, associations were examined between knowledge of AI, gender, medical experience, and other variables using IBM SPSS Statistics version 26 (IBM Corp., Armonk, NY, USA). P-value < 0.05 was considered statistically significant.

## Results

The overall responses of the medical students and doctors are presented in Table 2.

**Table 2:** Responses to the post-webinar questionnaire given by medical students and junior doctors This table presents the responses to the questionnaire sent to doctors and medical students. Chi-square statistical test was done. P < 0.05 was considered statistically significant.

Statements	Medical students (N = 212)	Doctors (N = 155)	P-value
Gender	Male: 126 (59.4%); female: 86 (40.6%)	Male: 90 (58.1); female: 65 (41.9%)	Not significant (P = 0.79)
I have never previously attended any webinar/lecture/course on Artificial Intelligence in healthcare.	177 (83.5%)	145 (93.5%)	P < 0.001
I strongly agree or agree with the statement “Artificial Intelligence will play an integral role in delivering healthcare services in the future.”	181 (85.4%)	92 (59.4%)	P < 0.001
I feel extremely unknowledgeable or unknowledgeable about the basic principles of Artificial Intelligence technology and its applications in healthcare.	156 (73.6%)	136 (87.7%)	P < 0.001
I feel extremely unknowledgeable or unknowledgeable about the limitations of Artificial Intelligence.	172 (81.1%)	132 (85.2%)	Not significant (P = 0.31)
I strongly agree or agree with the statement “Knowledge of Artificial Intelligence will be beneficial for your career as a doctor.”	179 (84.4%)	101 (65.2%)	P < 0.001
Top three resources that you have used the most to learn about Artificial Intelligence and its applications	Nothing (122, 57.5%), friends/colleagues (30, 14.2%), websites (27, 12.7%)	Nothing (90, 58.1%), journal articles (32, 20.6%), websites (14, 9%)	-
I feel extremely interested or interested in learning the principles of Artificial Intelligence and its applications in healthcare.	147 (69.3%)	80 (51.6%)	P < 0.001
I strongly agree or agree with the statement “Formal training is needed in medical schools and hospitals to teach about Artificial Intelligence and its applications in healthcare.”	125 (59%)	79 (50.1%)	Not significant (P = 0.13)
My medical school or hospital has given me extremely minimal or minimal advice about Artificial Intelligence and its applications.	171 (80.7%)	141 (91%)	P = 0.006
I strongly disagree or disagree with the statement “Artificial Intelligence may replace physicians in some specialties in the future.”	162 (76.4%)	105 (67.7%)	Not significant (P = 0.39)

Responses of medical students

Out of the total of 387 registrants, 212 students filled the post-event questionnaire (response rate = 54.8%). The majority identified as male (126, 59.4%) and were third-year medical students (74, 34.9%). Of note, the majority of the respondents (177, 83.5%) had never attended any webinar/lecture or course on AI in healthcare. However, the majority (181, 85.4%) either strongly agreed (50, 23.6%) or agreed (131, 61.8%) that AI will play an integral role in delivering healthcare services in the future.

Most of the respondents (156, 73.6%) either felt extremely unknowledgeable (73, 34.4%) or unknowledgeable (83, 39.2%) about the basic principles of AI and its applications in healthcare. Furthermore, a larger proportion of respondents (172, 81.1%) also felt either extremely unknowledgeable (142, 67%) or unknowledgeable (30, 14.2%) about the limitations of Artificial Intelligence.

The majority of the respondents (179, 84.4%) either strongly agreed (99, 46.7%) or agreed (80, 37.7%) with the statement “Knowledge of Artificial Intelligence will be beneficial for their career as a doctor.” However, the majority (122, 57.5%) had used nothing to learn about Artificial Intelligence and its applications. The most used resources to learn about AI were friends and colleagues (30, 14.2%), followed by websites (27, 12.7%) and journal articles and books (13, 6.1%).

Of note, the majority of the respondents (147, 69.3%) either felt extremely interested (76, 35.8%) or interested (71, 33.5%) in learning the principles of Artificial Intelligence and its applications in healthcare. Additionally, the majority of the respondents (125, 59%) either strongly agreed (45, 21.2%) or agreed (80, 37.7%) with the statement “Formal training is needed in medical schools and hospitals to teach about Artificial Intelligence and its applications in healthcare.”

However, the majority of the respondents (171, 80.7%) felt that their medical school either gave them extremely minimal advice (85, 40.1%) or minimal advice (86, 40.6%) in teaching about AI and its application. Of note, most of the respondents (162, 76.4%) either strongly disagreed (126, 59.4%) or disagreed (36, 17%) with the statement “Artificial Intelligence may replace physicians in some specialties in the future.”

Gender disparities among medical students

A significantly (P = 0.02) higher proportion of male respondents (27, 21.4%) had previously attended a webinar/lecture or course on AI in healthcare as compared with female respondents (8, 9.3%). A significantly (P = 0.001) higher percentage of female respondents (66, 76.7%) either felt extremely unknowledgeable or unknowledgeable about the basic principles of Artificial Intelligence technology and its applications in healthcare than male respondents (90, 71.4%). However, female respondents (66, 76.7%) were significantly (P = 0.004) more interested than male respondents (81, 64.3%) to learn about the principles of AI and its applications in healthcare.

Responses of doctors

Among 234 registrants, 155 doctors filled the post-event questionnaire (response rate = 66.2%). The majority identified as male (90, 58.1%) and had never attended any webinar/lecture or course on AI (145, 93.5%). Similar to the medical students’ perceptions, the majority of doctors felt that AI will play an integral role in delivering healthcare services in the future (92, 59.4%).

However, a higher proportion of doctors felt unknowledgeable about the principles (136, 87.7%) and limitations (132, 85.2%) of AI than medical students. A relatively lesser proportion of doctors (101, 65.2%) felt that AI will be beneficial for their career as a doctor as compared with medical students. The majority of doctors had used nothing (90, 58.1%) to learn about AI and felt that formal training should be given by their hospitals (79, 50.1%).

A relatively lesser proportion of doctors felt interested to learn more about AI (80, 51.6%) than medical students. The majority of doctors received minimal advice from their hospitals (141, 91%) and felt that AI will not replace doctors in some specialties in the future (105, 67.7%).

## Discussion

Due to limited resources and opportunities, students and doctors from less developed nations are usually at a disadvantage compared to those from developed countries to get access to new software or technologies. Lack of knowledge and gender disparities are clearly seen in our study, which further reflects the unmet needs of healthcare professionals, and more emphasis needs to be put on their upliftment. Formal training courses or extracurricular academic events can be started, especially for those who are interested, and further modifications can be made based on the feedback received.

A cross-sectional study conducted in a high-income country concluded that the majority of medical students do not feel properly equipped to work with AI tools but realize the rising relevance of AI in healthcare and would want to get further teaching on this emerging field [15]. A similar study in a lower middle-income country also concluded that the majority of medical students are not fully aware of AI [16]. These findings are comparable to the results of our study, in which the majority of students felt that they have limited knowledge about AI principles and applications.

Of note, as the majority of the doctors and medical students in our study felt that AI will play an integral role in delivering healthcare services in the future, it is of prime importance to teach them about the functions of AI. For example, AI findings are only as good as the input data, and its results may not accurately represent the minority population if the initial input database is skewed toward a certain gender or ethnicity [17]. Furthermore, limitations and ethical concerns related to AI shall also be emphasized so that accurate interpretation of high-quality findings is used for future policy development. Overreliance on AI may lead to automation bias. Hence, sophisticated regulatory oversight and legal frameworks are needed for the sustainable usage of AI in healthcare for physicians and patients [9]. More work needs to be done to create awareness about both the abilities and limitations of AI among medical professionals as found in our analysis.

To tackle the unmet need of lack of knowledge despite high interest in AI, a multidisciplinary, integrated approach may be used by enrolling interested doctors and students into electives or relevant courses [11]. The role of medical universities and hospitals to boost the cognizance about AI is important especially since the majority of the respondents felt that they have received minimal teaching about AI from their schools/hospitals. It will also further help in the standardization of learning materials so that authentic information is disseminated. Currently, the majority of the respondents who participated in our study are learning about AI from their friends/colleagues and websites, and nothing can be surely said about the accuracy of these sources.

In our study, doctors felt less interested than medical students to learn more about AI and received minimal advice from their training institutions, which may be due to a lack of opportunities. However, they also felt that AI will play an integral role in delivering medical services in the future, which may reflect that early teaching about AI in the medical curriculum shall be inculcated to spark an interest in this upcoming field. Several studies had conflicting findings of the perception of medical students about whether AI will replace humans in a medical specialty, here radiology [15,18-20]. Although, in our study, the majority of students agree that AI will not replace physicians in the future, it is recommended to pursue positive efforts in spreading awareness about AI so that misinformation and human hesitancy can be reduced. The limitations of our study include recall bias and self-reporting errors as the present was a questionnaire-based cross-sectional study.

## Conclusions

In our study, gender disparities and a clear lack of knowledge have been found in medical students and doctors regarding AI in healthcare. Formal training courses to teach about AI should be focused on to facilitate coherent and scientifically supported dissemination of knowledge in medical schools and hospitals. Further large-scale studies are needed to understand the perception and attitude of medical students and doctors regarding AI to steer policy development and medical education curriculum changes to spark an interest in emerging technologies and drive innovation.
